# A comparative study of renal function in male and female spiny mice – sex specific responses to a high salt challenge

**DOI:** 10.1186/2042-6410-4-21

**Published:** 2013-12-10

**Authors:** Hayley Dickinson, Karen M Moritz, Michelle M Kett

**Affiliations:** 1 The Ritchie Centre, Monash Institute of Medical Research, Monash University, Clayton, VIC, Australia; 2 School of Biomedical Sciences, University of Queensland, Brisbane, Australia; 3 Department of Physiology Monash University, Clayton, Australia

**Keywords:** Kidney, *Acomys cahirinus*, NaCl

## Abstract

**Background:**

There is a significant body of evidence to suggest that hormone levels, receptor density and structural differences between males and females can significantly alter renal hemodynamics. We compared the renal hemodynamic and excretory profile of female and male spiny mice under baseline conditions and in response to a high-NaCl diet.

**Methods:**

Adult male and female spiny mice were fed either a normal or high salt diet for 7 days. Renal excretory profile was obtained from 24 h urine samples, and renal hemodynamic measurements using anaesthetised renal clearance techniques. Kidneys were excised, weighed and frozen for qPCR analysis.

**Results:**

Under basal conditions, conscious and anaesthetised renal functions were similar between male and female spiny mice when adjusted for body weights. Male and female spiny mice on the high-NaCl diet had significantly greater GFR than sex matched controls (P_DIET_ < 0.001). However the magnitude of the effect of salt was sex dependent (P_SEX_ < 0.001; P_INT_ < 0.01). Male spiny mice showed a greater increase in GFR (84% higher than normal salt males) compared to females (33% higher than normal salt females), despite similar increases in renal plasma flow. In response to 7 days of high salt diet, female spiny mice showed a greater increase in 24-hour water consumption (45% more) and urinary output (50% more) compared to males (P_INT_ < 0.01). These sex differences could not be explained by differences in renal expression of the V2R or AQP3 channel.

**Conclusion:**

These studies have identified major differences between male and female spiny mice in their renal response to a high-NaCl load suggesting that renal hemodynamics may be differentially regulated for the sexes.

## Background

Rodent species such as the rat and mouse have been, and will continue to be, crucial in our understanding of human health and disease. However in the fields of fetal programming and suboptimal pregnancy, they have a distinct drawback in their utility as models of human pregnancy. Typical rodents have relatively short gestation periods (18-21 days), giving birth to large litters of offspring in which development of major organs, including the kidney, is incomplete at birth and continues well into the neonatal period. The spiny mouse (*Acomys cahirinus*) is a nocturnal species native to regions of Egypt and Israel, where it inhabits sandy deserts and rocky terrains giving birth to 1–3 young [1]. In stark contrast to typical rodents, organogenesis of the spiny mouse kidney [2], liver [3], lung [4] and various brain regions [5-7] are completed during the 39-day period of fetal life, and thus spiny mice are born at a developmental stage comparable to the human newborn. Indeed the spiny mouse is the only known rodent species to complete nephrogenesis before birth [2]. Owing to this, and the comparable endocrinology of pregnancy [8], the spiny mouse is now being utilized as a preclinical model of sub-optimal human pregnancy. Using this species, we have developed robust and repeatable models of birth asphyxia [9-13], maternal viral infection [14] and excess maternal glucocorticoids [15-17]. These studies of sub-optimal pregnancy in the spiny mouse have highlighted numerous sex-specific differences including placental gene expression and structural development [15,16,18], postnatal growth and survival after birth asphyxia [11] and susceptibility to adult renal dysfunction [17] after excess maternal glucocorticoid exposure in utero.

There is a growing body of evidence to suggest that hormone levels, receptor density and structural differences between males and females can significantly alter renal hemodynamics [19,20]. These differences may be implicated in the sexually dimorphic incidence of diseases such as diabetic nephropathy, acute and chronic ischemic renal failure, aging, and susceptibility to adult renal disease following an adverse intrauterine environment [21]. It is generally considered that males drink less, and are able to concentrate their urine more than females contributing to the higher risk of urolithiasis [22-24], however the mechanisms are poorly described [20]. The current study aimed to determine whether fundamental differences in renal function exist between the sexes in adult spiny mice. Secondly, as differences are often only exposed following a challenge, we also examined the response of male and female spiny mice to a high salt diet. We hypothesized that spiny mice will exhibit sex specific responses to a high-NaCl load and that this will be reflected in differences in renal excretory and hemodynamic profiles.

## Methods

### Animals

All experiments were approved in advance by Monash University School of Biomedical Sciences Animal Ethics Committees and conducted in accordance with the Australia Code of Practice for the Care and Use of Animals for Scientific Purposes. Adult (13–15 week old) male (n = 26) and female (n = 25) spiny mice used in this study were obtained from our own laboratory colony as previously described [2].

### Conscious renal function

Trained spiny mice (n = 16 male, 15 female) were placed in metabolic cages to obtain baseline 24 h urine samples on a normal salt diet (0.25% w/w NaCl; Specialty Feeds, WA, Australia). Spiny mice were then allocated to receive a high salt diet (10% w/w NaCl; Specialty Feeds, WA, Australia) or remain on normal salt chow (5/sex/diet), and measurements repeated on days 3 and 7. A high, 10% w/w, NaCl diet was chosen as the spiny mouse is a desert species and high-salinity vegetation or invertebrates are often the only available source of nutrients and water [25]. Metabolic cage data collected on days 3 and 7 did not differ; therefore only data collected on day 7 are presented. Further, the data obtained on days 3 and 7 for normal salt male and female time controls did not differ from baseline values and are not presented.

### Urine collection and analysis

Body mass, water and food consumption, and faeces and urine excretion were recorded for all spiny mice. Urine was collected and frozen for subsequent analysis of urinary electrolytes. All urine samples were analysed for sodium (Na), chloride (Cl), potassium (K) and urea by spectrophotometry (Synchron CX5CE Delta; Beckman Coulter, Fullerton, CA, USA). Osmolality of urine and plasma samples was measured by freezing point depression (Advanced Osmometer 2020; Advanced Instruments, Needham Heights, MA). Protein concentration of urine samples was measured by BCA Protein Assay (Kit#23225, Thermo Scientific, Australia).

### Anaesthetised renal function

Spiny mice (n = 5/sex/diet) were maintained on either a normal salt diet (0.25% w/w NaCl; Specialty Feeds, WA, Australia) or placed on a high salt diet (10% w/w NaCl) for 7 days prior to anaesthetised renal function measurements (data obtained from males in this cohort has been published in part [26]). Briefly, spiny mice were anaesthetised (Isoflurane mixed with 40% O_2_, 60% N_2_; 4.5% induction, 2.5–2.8% maintenance; Rhodia Australia P/L, Notting Hill, VIC, Australia) and placed on a heated pad to maintain body temperature at 37.5°C. A catheter (tapered SV-35 tubing) was inserted into the right carotid artery for continuous measurement of blood pressure and heart rate (HR), and to obtain a terminal arterial blood sample. The left jugular vein was catheterized (PE-10 tubing) for infusion of a 1% bovine serum albumin (BSA) solution containing ^3^H-inulin (5.58 μCi/mL) and ^14^C-paraaminohippurate (PAH; 1.7 μCi/mL) for estimation of glomerular filtration rate (GFR) and effective renal plasma flow (ERPF) respectively by renal clearance methods [26,27]. The bladder was catheterised to allow the collection of urine, and the mice were allowed to equilibrate for 1 h. Following equilibration, urine was collected over two timed 15 min periods after which an arterial blood sample was taken. Animals were killed at the completion of the renal function measurements and kidney samples frozen and fixed for further analysis.

### Real-time PCR

Real-time PCR primer design for Aquaporin 3 (AQP3; GenBank NM_004925.4, NM_016689.2) and vasopressin type 2 receptor (V2R; GenBank NM_019404.2, NM_019136.1, NM_001146151.1) was performed as previously described [15]. Briefly, for each gene of interest, where possible the cDNA sequences for *Mus musculus*, *Rattus norvegicus*, and *Homo sapiens* were aligned, and primers were designed based on identified regions of high homology. Optimizing PCR assays were conducted in order to test the specificity of the primer sets, which were found to amplify products of the expected sizes (data not shown). Primers for other aquaporin’s could not be designed for the spiny mouse because of lack of sufficient sequence homology between other species.

RNA was extracted from whole adult kidneys using the commercially available RNeasy Midi kit (Qiagen, Australia) and samples (1 ug RNA) were reverse transcribed to form cDNA. Quantitative real-time PCR (qPCR) cycling conditions for all genes consisted of an initial denaturation step of 95°C for 10 min, followed by 40 cycles consisting of 95°C for 15 sec and 60°C for 1 min. A dissociation curve was also performed at the end of each run. Samples were run in duplicate for each gene of interest. A comparative CT (cycle of threshold fluorescence) method was used for analysis with 18S used as an endogenous reference [15]. To calculate the relative expression levels in each sample, the CT value for 18S was subtracted from the CT value of the gene of interest to give a ΔCT value. The mean ΔCT value of the saline group was then subtracted from each individual sample to give a ΔΔCT. This number was then inserted into the formula 2^-ΔΔCT^ to give the expression level relative to the mean of the male normal salt diet group. The assay was run twice for each gene of interest.

### Data analysis and statistics

Data are presented as means ± SEM. Two-way ANOVA (repeated where appropriate), with diet (P_DIET_) and sex (P_SEX_) as the fixed factors, was used to determine whether the impact of the salt diet on renal variables were different between males and females. P_INT_ refers to an interaction effect between diet and sex. Bonferoni post-hoc analysis was used where stated. P values of ≤0.05 were considered statistically significant.

## Results

### Conscious renal function

Male spiny mice were approximately 14% heavier than age-matched females at the time of baseline measurements taken on a normal salt diet. When corrected for body weight, water and food intake and urine excretion were similar between the sexes (Table 1). The urinary excretion of sodium, urea, osmolites and urine osmolality were also similar between male and female spiny mice when corrected for bodyweight (Table 1).

**Table 1:** 24 hour renal function in male and female spiny mice

	**Male**	**Female**
**Body Weight (g)**	36.6 ± 0.7	32.0 ± 0.6*
**Water Intake (ul/gBW)**	101.8 ± 6.3	112.2 ±14.4
**Urine Volume (ul/gBW)**	37.2 ±3.1	35.3 ± 6.5
**Food Intake (mg/gBW)**	86.6 ± 4.1	79.9 ±7.1
**Na**^**+ **^**Excretion (umol/g BW)**	2.52 ± 0.26	2.46 ± 0.40
**Urea Excretion (umol/g BW)**	53.2 ± 3.2	45.4 ± 7.1
**Osmolality (mOsmol/kg H**_**2**_**O)**	2546 ± 419	2247 ±286
**Osmolar Excretion (uosmol/g BW)**	90.4 ± 13.2	70.6 ± 11.6
**n**	16	15

Values are mean ± SEM. *P < 0.001.

**Figure 1:**
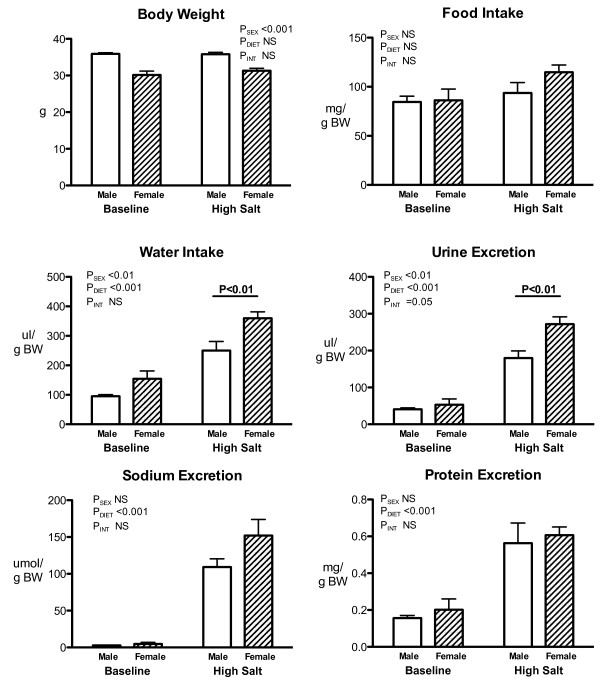
**Conscious renal function - 24 h conscious renal function in male (open bars) and female (hatched bars) spiny mice before (baseline) and after 7 days of high salt diet.** n = 5/sex/diet, P-values by Two-way Repeated ANOVA with sex and diet as the fixed factors.

The 7 days of high salt diet did not effect bodyweight or food intake in male or female spiny mice (Figure 1). Male and female spiny mice showed a significant increase in water consumption and urine output in response to the high salt diet (P_DIET_ < 0.001; Figure 1). There was a significant effect of sex on both water consumption (P_SEX_ = 0.005) and urine excretion (P_SEX_ = 0.007). Post-hoc analysis showed that whilst baseline water consumption and urine output between the sexes were not significantly different, on a high salt diet female spiny mice consumed 45% more water and excreted 50% more urine than males (P < 0.01; P < 0.05). Male and female spiny mice exhibited a similar, marked increase in urinary Na, and protein, on the high salt diet, however there was no difference in the response of the sexes to the high salt diet (P_DIET_ < 0.001; P_SEX_ = NS; Figure 1). Total osmolar excretion increased, and urine osmolality fell in response to the high salt diet, an effect that was similar between the sexes (Data not shown). Urinary excretion of K was similar between the sexes and did not change with high salt diet (Data not shown).

### Anaesthetised renal function

Anaesthetised renal function values for male and female spiny mice following 7 days of high salt or normal salt diet are described in Table 2. Anaesthetised MAP, ERPF, and RVR values were similar between the sexes (Table 2). ERPF was approximately 55% greater in males and females on the high salt diet compared to normal salt controls (P_DIET_ < 0.001). MAP was mildly but significantly (P_DIET_ = 0.04) greater in high salt compared to normal salt groups resulting in a markedly lower RVR in high salt compared to normal salt groups (P_DIET_ = 0.002; Table 2). There was however no difference in the response of the sexes to the high salt diet for ERPF, MAP or RVR (P_INT_ = NS; Table 2).

**Table 2:** Anaesthetised renal function and PCR analysis of male and female spiny mice on normal or high salt diets

	**Male**		**Female**		**Statistics**		
	**Normal salt**	**High salt**	**Normal salt**	**High salt**	**P**_**SEX**_	**P**_**DIET**_	**P**_**INT**_
**Mass**							
**Pre-diet Body Mass g**	35.8 ± 0.4	35.7 ± 0.9	33.5 ± 1.0	31.8 ± 0.7	**=0.001**	0.29	0.35
**Post-diet Body Mass g**	36.8 ± 0.6	37.1 ± 0.6	33.9 ± 0.8	32.0 ± 0.9	**<0.001**	0.29	0.16
**Change in Body Mass g**	1.0 ± 0.3	1.4 ± 0.6	0.4 ± 0.4	0.2 ± 0.3	0.052	0.84	0.46
**Kidney Mass mg**	268 ± 11	295 ± 10	263 ± 9	285 ± 8	0.42	**=0.01**	0.80
**KM: BM Ratio mg:g**	7.27 ± 0.23	7.97 ± 0.18	7.75 ± 0.16	8.95 ± 0.33	**=0.005**	**<0.001**	0.29
**Renal & Cardiovascular Measures**							
**MAP mmHg**	62.0 ± 1.5	67.4 ± 1.7	60.3 ± 2.0	62.4 ± 1.6	0.07	**=0.04**	0.36
**GFR μL/min/gBM**	3.66 ± 0.12	6.63 ± 0.34	3.43 ± 0.28	4.83 ± 0.51	**=0.005**	**<0.001**	**=0.025**
**ERPF μL/min/gBM**	11.60 ± 0.54	18.36 ± 1.38	11.51 ± 1.80	17.61 ± 1.38	0.76	**<0.001**	0.81
**RVR mmHg/μL/min/gBM**	3.03 ± 0.10	2.12 ± 0.17	3.35 ± 0.48	2.09 ± 0.15	0.62	**=0.002**	0.55
**Filtration Fraction%**	32 ± 2	37 ± 3	32 ± 3	27 ± 1	0.07	0.95	**=0.048**
**Renal PCR**							
AQP3	1.00 ± 0.13	0.72 ± 0.14	0.75 ± 0.14	0.73 ± 0.09	0.46	0.22	0.28
V2R	1.00 ± 0.15	0.79 ± 0.20	0.67 ± 0.12	0.91 ± 0.14	0.32	0.64	0.28

Values are means ± SEM. n = 6 per sex on normal salt diet, n = 5 per sex on high salt diet. *NS*, non significant, g; gram, *BM*, body mass, *KM*, kidney mass, *MAP*, mean arterial pressure, *GFR*, glomerular filtration rate, *ERPF*, effective renal plasma flow, *RVR*, renal vascular resistance. Data were analysed by Two way ANOVA.

High salt diet and sex had significant effects on GFR. Male and female spiny mice on the high salt diet had significantly greater GFR than sex matched controls (P_DIET_ < 0.001). However the magnitude of the effect of salt was sex dependent (P_SEX_ = 0.005; P_INT_ = 0.025; Table 2). The effect of high salt diet was greatest in males, with the GFR of high salt males 84% higher than normal salt controls whereas females on high salt had GFR values that were only 33% higher than normal salt controls (P_INT_ = 0.025; Table 2). As a result of the sexually dimorphic response of GFR but not ERPF to high salt diet, filtration fraction of male spiny mice on the high salt diet was higher than normal salt controls, whereas in females, filtration fraction on the high salt diet was lower than controls (P_INT_ = 0.048; male, 32-37%; female, 32-27%; Table 2).

Body mass was greater in male than female spiny mice (P_SEX_ < 0.001; Table 2) however there was no effect of salt diet on body mass or the change in body mass over the 7 days of dietary treatment in either sex. There was no sex difference in total kidney mass, thus kidney to body mass ratio was higher in females compared to males (P_SEX_ < 0.001; Table 2). High salt diet had a significant impact on kidney mass leading to greater kidney mass (P_DIET_ = 0.01; Table 2) and kidney to body mass ratios (P_DIET_ < 0.001; Table 2) compared to normal salt controls in males and females. Heart rate (male normal salt (NS) 472 ± 8, high salt (HS) 464 ± 16; female NS 492 ± 10, HS 476 ± 14 bpm) and plasma osmolality (male NS 332 ± 6, HS 361 ± 17; female NS 343 ± 22, HS 348 ± 11 mosmol/kg H_2_O) did not differ between the sexes or salt groups.

### Real-time PCR

The renal expression of AQP3 and V2R did not differ between male and female spiny mice and was not effected by the level of dietary salt (Table 2).

## Discussion and conclusions

The spiny mouse is a unique species amongst rodents due to the fact that nephrogenesis, in addition to development of other organs, is completed prior to birth [2]. As such it has a distinct advantage over other rodents as a preclinical model of human pregnancy. We have detected distinct sexual dimorphic responses of the fetal/placental unit and the kidney specifically, to sub-optimal pregnancy [16,17]. However we do not have a good understanding of basic sex differences in renal physiology in adult spiny mice and how they compare with other rodent species and, importantly, humans. Our study aimed to determine whether there were sex differences in renal function of adult spiny mice under baseline conditions and in response to the challenge of a 7-day high salt diet. We found there was little difference in basal renal function between male and female spiny mice when differences in body weight were accounted for. However we detected a dramatic, sexually dimorphic response when mice were challenged with a high salt diet. These findings suggest that males and females respond to a high-salt load by different mechanisms, and may contribute to the greater susceptibility to renal disease in males.

### Basal sex differences in renal function

Under basal conditions we found that both conscious and anaesthetised renal function were similar between male and female spiny mice. When corrected for bodyweight, 24-hour food and water intake, urine production, and the excretion of sodium and osmolytes were similar in male and female spiny mice. In anaesthetised experiments we again found that renal function was similar with renal plasma flow, renal vascular resistance and filtration fraction not different between the sexes. Although 2-way ANOVA detected a significant sex effect for GFR, this result was likely driven by the large effect of salt in males. Indeed on normal salt, GFR in males was only 7% greater than females. Thus under controlled, low dietary salt conditions, male and female spiny mice show similar renal function relative to bodyweight.

Evidence from human and animal studies suggests that there are significant differences in renal physiology between the sexes due to differences in vascular responsiveness and the level of expression and action of renal transporters [19]. For example, studies in rats have shown that whole kidney GFR and single nephron GFR are higher in males than females due to a higher renal plasma flow and lower renal vascular resistance, that result in sustained hyperfiltration in males [28-30]. However, consistent with our anaesthetised renal function findings, most studies have demonstrated that when indices of renal function have been factored for the higher bodyweight or kidney weights of males, these sex differences are greatly diminished [29]. Studies in rodents and human also suggest that females drink more and excrete a greater volume, of less concentrated, urine [20,22-24]. However in a review of the human literature, Perucca et al. [20] noted that whilst 24 h urine volumes in 4 of 9 studies were a modest 10-14% lower in males, in the remaining 5 studies urine volumes were similar between the sexes as in our study of the spiny mouse.

### Sex-specific renal adaptations to a high salt load

When challenged with a high salt load, significant differences in renal function were observed between male and female spiny mice. Female spiny mice fed a high salt diet for 7 days demonstrated a significantly greater increase in water consumption and urine production when compared to male spiny mice. In contrast, whilst both female and male spiny mice on a high salt diet had significantly greater GFR, renal plasma flow and MAP compared to control spiny mice, the increase in GFR of the male spiny mice on the high salt diet was significantly greater than female spiny mice, despite similar increases in renal plasma flow. The major finding therefore of these studies is that there are sex specific differences in the renal response of spiny mice to a high-salt load.

Consistent with our findings, studies in humans have found that males excrete high osmolar loads by increasing urinary concentration rather than increases in urine volume [20]; a feature that has been linked to the higher rates of urolithiasis in males [19,20]. The mechanisms underlying the higher urine excretion and drinking rates of female compared to male spiny mice on the high salt diet are unclear but may be due to sex differences in levels of, and sensitivity to, vasopressin. Some studies have reported higher plasma and urinary vasopressin levels in men [20]. These higher levels of vasopressin in males may be due to greater secretion as several studies have found that vasopressin secretion in males is more sensitive to osmotic stimuli (e.g., hypertonic saline infusion); see [20,31-33]. Further, levels of V2 receptors have also been found to be higher in male rats [33]. Sex steroids are likely to contribute to these differences with Longhurst and colleagues showing that estradiol treatment of ovariectomized rats caused significant increases in water consumption and urine excretion when compared to ovariectomized and sham-operated rats [34]. The authors concluded that estrogen attenuates the antidiuretic effect of vasopressin, a finding that was further supported by Wang et al. [24], who found that the antidiuretic activity of vasopressin was effected by the estrous cycle.

We investigated whether the renal expression of vasopressin receptors and aquaporins would differ between the sexes providing a mechanism for the different sensitivity of the sexes to high salt. Unfortunately, due to low species homology between the aquaporins (in regions of the genes appropriate for primer design) we were unable to examine AQP1, which is directly involved in urinary concentration in the proximal tubule and descending thin limb, or AQP2 which is the predominant vasopressin-regulated water channel expressed in the connecting tubule and collecting duct [35]. We were able to examine AQP3 which is primarily expressed in the collecting duct where it is reported to have a role in osmotically driven water absorption across the collecting duct epithelium with AQP4 [36], however the expression of AQ3 did not differ between the sexes. Despite findings in rats that males have higher expression of V2R compared to females [33], we found that the expression of V2R did not differ between the sexes suggesting that it is not a difference in transcription of the vasopressin receptor that mediates the sex differences observed here. Indeed, further work to examine other aquaporins as well as plasma vasopressin levels between the sexes is warranted.

Our study has identified a sexual dimorphic renal hemodynamic response to high salt diet. On the high salt diet both sexes demonstrated a fall in renal vascular resistance and concomitant increase in renal plasma flow. This drop in resistance also facilitated an increase in GFR, however there was a significant difference between the sexes in the magnitude of the response. The GFR of high salt males was 84% higher than normal salt controls, whereas females on high salt had GFR values that were only 33% higher than normal salt controls. An enhanced renal function response to high salt diet has also been reported in men. Interestingly, whilst young men showed increases in GFR and renal plasma flow in response to chronic salt-loading [37], women (luteal or follicular phase) did not show these increases in renal function [38]. Sex steroids, sexual dimorphism in the renin angiotensin and endothelin systems have been implicated in the differential response to high salt diet [39,40].

The higher GFR in males compared to females, with no difference between the sexes for RVR, suggests that there was a significant difference in the ratio of pre to post-glomerular resistance changes between the sexes. Significantly the data suggests that the males showed significantly greater preglomerular vascular dilation than female spiny mice. As this would indicate significantly greater glomerular capillary pressure in males versus females, it would suggest that prolonged, chronic exposure to high salt would lead to greater hyperfiltration induced injury in males compared to females. Consistent with this hypothesis, studies in humans and animal models have shown that males show an accelerated age-related decline in renal function and are more sensitive to acute and chronic renal injury [19,29,41].

In summary, our studies have highlighted that when adjusted for bodyweight, renal excretory and hemodynamic function of the spiny mouse are similar between the sexes under control conditions. However we identified a sexually dimorphic response to the physiological challenge of a high salt load. Male spiny mice were able to excrete the salt load in a reduced urine volume compared to females. Further, male spiny mice showed an exaggerated rise in GFR on the high salt load consistent with greater preglomerular dilatation. These responses may contribute to the greater susceptibility of males to renal disease.

## Abbreviations

GFR: Glomerular filtration rate; ERPF: Effective renal plasma flow; ERBF: Effective renal blood flow; RVR: Renal vascular resistance; NaCl: Sodium chloride; AQP: Aquaporin; V2R: Vasopressin type 2 receptor; MAP: Mean arterial pressure; HS: High salt; NS: Normal salt; bpm: Beats per minute; ANOVA: Analysis of variance; CT: Cycle threshold; BSA: Bovine serum albumin; PAH: Paraaminohippurate; PCR: Polymerase chain reaction; RNA: Ribonucleic acid.

## Competing interests

The authors do not have any competing interests to declare.

## Authors’ contributions

HD contributed to study conception and design, carried out animal work and performed all urine analysis and qPCR. KMM contributed to study conception and design. MMK contributed to study conception and design, performed animal work with HD and prepared the final results and performed statistical analysis. All authors read and approved the final manuscript.
